# Socioeconomic differences in childhood BMI trajectories in Belarus

**DOI:** 10.1038/s41366-018-0042-0

**Published:** 2018-02-28

**Authors:** Rita Patel, Kate Tilling, Debbie A Lawlor, Laura D Howe, Rachael A Hughes, Natalia Bogdanovich, Lidia Matush, Emily Nicoli, Emily Oken, Michael S Kramer, Richard M Martin

**Affiliations:** 1 Population Health Sciences, Bristol Medical School, University of Bristol, Bristol, UK; 2 MRC Integrative Epidemiology Unit, University of Bristol, Bristol, UK; 3 The National Research and Applied Medicine Mother and Child Centre, Minsk, Belarus; 4 Division of Chronic Disease Research Across the Lifecourse, Department of Population Medicine, Harvard Pilgrim Health Care Institute and Harvard Medical School, Boston, MA USA; 5 Departments of Pediatrics and of Epidemiology, Biostatistics and Occupational Health, McGill University Faculty of Medicine, Montreal, Canada; 6 National Institute for Health Research Bristol Biomedical Research Unit in Nutrition, Bristol, UK

## Abstract

**Objective:**

To examine associations of parental socioeconomic position with early-life offspring body mass index (BMI) trajectories in a middle-income country.

**Subjects:**

Overall, 12,385 Belarusian children born 1996–97 and enrolled in a randomised breastfeeding promotion trial at birth, with 3–14 measurements of BMI from birth to 7 years.

**Methods:**

Cohort analysis in which exposures were parental education (common secondary or less; advanced secondary or partial university; completed university) and occupation (manual; non-manual) at birth, and the outcome was BMI *z*-score trajectories estimated using multilevel linear spline models, controlling for trial arm, location, parental BMI, maternal smoking status and number of older siblings.

**Results:**

Infants born to university-educated mothers were heavier at birth than those born to secondary school-educated mothers [by 0.13 BMI *z*-score units (95% confidence interval, CI: 0.07, 0.19) for girls and 0.11 (95% CI: 0.05, 0.17) for boys; equivalent for an infant of average birth length to 43 and 38 g, respectively]. Between the ages of 3–7 years children of the most educated mothers had larger BMI increases than children of the least educated mothers. At age 7 years, after controlling for trial arm and location,  children of university-educated mothers had higher BMIs than those born to secondary school-educated mothers by 0.11 *z*-score (95% CI: 0.03, 0.19) among girls and 0.18 (95% CI: 0.1, 0.27) among boys, equivalent to differences in BMI for a child of average height of 0.19 and 0.26 kg/m^2^, respectively. After further controlling for parental BMI, these differences attenuated to 0.08 *z*-score (95% CI: 0, 0.16) and 0.16 *z*-score (95% CI: 0.07, 0.24), respectively, but changed very little after additional adjustment for number of older siblings and mother’s smoking status. Associations were similar when based on paternal educational attainment and highest household occupation.

**Conclusions:**

In Belarus, consistent with some middle-income countries, higher socioeconomic position was associated with greater BMI trajectories from age 3 onwards.

## Introduction

Overweight and obesity among school-aged and adolescent children have increased among all countries, including those of Eastern Europe [1]. These countries have distinctly differing political, social and economic histories compared to Western developed countries. Child obesity is a major public health concern, as obesity tracks from childhood to adulthood [2] and causes major physical and psychosocial problems, both in the short and long term [3]. Differences in the prevalence of childhood overweight and obesity between socioeconomic groups may contribute to life-long inequalities in health. Systematic reviews of cross-sectional studies show that, in countries with low levels of economic development, obesity is more common among children from more affluent or more educated families [4, 5]. Although in economically developed (Western) countries, obesity is more common among less affluent or less educated families [4, 6]. These cross-sectional analyses cannot indicate the age at which early-life socioeconomic differences in obesity emerge, nor changes in the association with age [5]. By understanding when differential growth rates arise between socioeconomic groups, we can indicate the important period during childhood when socioeconomic differences first emerge, and contrast these findings with those of other countries.

Few longitudinal studies have been published from low- and middle-income countries. In particular, countries from the former Soviet Union [7–9] are instructive, because they have undergone periods of rapid recent change. Some previous longitudinal studies of weight or adiposity gain have examined trajectories in different measures that depend on age. For example, ponderal index (PI, kg/m^3^) was conventionally reported from birth to 2 years versus body mass index (BMI, kg/m^2^) from 2 years upward [10]. Others have reported outcomes that are not easily interpretable in terms of actual weight, e.g., coefficients determined as functions of age [11]. Still others do not account for length or height; if weight alone is the outcome, then any observed differences could be due to differences in stature, which influences weight [11].

Our study is based on a large, contemporary cohort from Belarus, a middle-income [12], former Soviet country with high adult literacy, low child mortality rates, and low income inequality [13] but high adult mortality rates, particularly from cardiovascular disease [14]. In an earlier publication, we observed an overall prevalence of overweight or obesity of 10% among girls and 9% among boys at mean age 6.5 years; children with parents of high education or non-manual occupations were more likely to be overweight or obese, compared to those of low education or manual occupations [15]. In addition, children of the most educated or non-manual parents were more likely to be taller at all ages [16, 17].

In this paper, we model longitudinal BMI trajectories by socioeconomic position, using multilevel linear spline models and repeated measures of BMI. Our method yields a simple continuous index from birth to age 7 years that accounts for length or height and age, with easily interpretable coefficients in terms of BMI *z*-scores. Our approach allows a straightforward comparison between socioeconomic groups and overcomes several limitations of previous studies. We examine the age at which socioeconomic differences in BMI *z*-score trajectories emerge, and the extent to which these early-life differences are explained by factors related to childhood adiposity and socioeconomic position: parental size, maternal smoking status and number of older siblings [18–21]. We focus on identifying the emergence of early-life differences in BMI by socioeconomic position in order to contrast the pattern with those found in other settings.

## Subjects and methods

### Study design and participants

The study cohort is based on 17,046 Belarusian children and their mothers, recruited in 1996–97 into the cluster-randomised Promotion of Breastfeeding Intervention Trial (PROBIT) [22] during their postpartum hospital stay. Briefly, 31 maternity hospitals and one each of their affiliated polyclinics (outpatient clinics for routine health care) were randomly assigned to participate in a breastfeeding promotion intervention or to continue their prevailing practices. Each hospital-polyclinic site was classified by geographical location (East or West of Belarus and urban or rural setting). Inclusion criteria specified that the mothers were healthy and initiated breastfeeding. At birth, infants were healthy, full-term (≥37 weeks gestation) singletons. Infants also weighed ≥2500 g at birth; and had an Apgar score of ≥5 at 5-min. Study staff estimated that only 1–2% of eligible women declined participation in the trial [23].

### Exposures

During their postpartum stay, mothers reported parental education and occupation [22]; we recoded educational status from seven original categories to three categories: primary only, incomplete or common secondary; advanced secondary or partial university; and completed university, with ‘unknown’ coded as missing. The parents of the PROBIT children were born and grew up during the Soviet era, and schooling (including university education) was widely available and free. We classified each parent’s occupation as either manual (e.g. farmer) or non-manual (e.g. service worker); the highest household occupation was used in our analysis [15]. By way of context, the average monthly wage in the agricultural sector in Belarus in 1997 was 1,405,130 Belarusian roubles (Br) ($54 USD) and in the community, social and personal services sector was 2,075,092 Br ($80 USD) [24]. Parents who reported they were pupils, students, unemployed, or housewives, or whose occupation was unknown, were coded as missing, because the relationship of these categories to usual socioeconomic position is unclear.

### Measurement of BMI

Infant birth weight and length were abstracted from medical records at the start of the study [22]. At scheduled study visits when the infant was aged 1, 2, 3, 6, 9 and 12 months, study paediatricians recorded weight and length; home visits were made when polyclinic visits were missed [22]. As differences in weight and length were not initially major hypotheses of PROBIT, no attempt was made to standardise these measurements in infancy. In 2002–05, at a median age of 6.5 years (interquartile range, IQR: 6.5–6.7; range: 5.6–8.5 years), 13,889 children (82% of those randomised) were examined at a research visit by one of 38 polyclinic paediatricians, who received special training in anthropometry using a standardised protocol. Weight (using an electronic digital scale, Bella 840; Seca Corporation, Hamburg, Germany) and standing height (using a stadiometer with a movable headboard, Medtechnika, Pinsk, Belarus) were measured twice and the average used [25]. An audit of 190 children at 5.3–32.6 (mean 17.7) months after the initial 6.5-year research visit showed a test-retest correlation of 0.89 for BMI. For the intervening period from age 12 months to 6.5 years, the study paediatrician retrospectively abstracted weight and height data for each child that had been recorded in the polyclinic records during routine check-ups. We calculated BMI-for-age *z*-scores with the *zanthro* command in STATA [26], using the WHO reference. The WHO BMI reference is based on two different methodologies using different reference data sets across the ages included in our study: longitudinal growth standard data for 0–5 years for healthy breastfed infants, and cross-sectional reference data after age 5 [27–29]. This reference was chosen as the most appropriate for this population, as it provides a continuous scale for BMI-for-age *z*-scores from birth to 7 years and is widely used, thus facilitating comparison of our findings with those from other studies. Those individuals with implausible BMI *z*-scores (greater than 4 standard deviations from the mean) were excluded (259 measurements for 198 children).

### Covariables

At recruitment, mothers reported the number of older children living in the household and their smoking status during pregnancy [22]. We categorised the number of older children in the household as 0, 1 or 2 or more. Current maternal smoking status was recorded by study paediatricians on scheduled study visits when the infant was aged 1, 2, 3, 6, 9 and 12 months [22]. The parent or guardian (in most cases the mother) who accompanied the child at the 6.5-year follow-up visit reported weight, height and current smoking status for herself and her partner. All occasions when maternal smoking was reported (i.e. during pregnancy, at clinic visits during the first-year of follow-up and at 6.5 years postpartum) were recoded to any versus none from the number of cigarettes smoked per day and combined to create a measure of ‘maternal smoking’ categorised as unknown, never or ever. The institutional review board of the Montreal Children’s Hospital approved both the original PROBIT trial and the 6.5-year follow-up; the participating parent or guardian signed consent forms in Russian at each phase.

## Statistical methods

### Summarising individual BMI *z*-score trajectories

We chose age 7 years (84 months) as our upper age cut-off for this study, as this was the oldest age with large numbers of measurements to describe the BMI *z*-score trajectory by socioeconomic groups and enabled us to identify the earliest age at which differences emerged. The PROBIT cohort is rich in data from birth to 7 years, with 3–14 repeated measures of BMI during this period for each child. Those with fewer visits did not attend all of the eight scheduled clinic visits, whereas those with many visits attended all the scheduled visits and, in addition, had weights and heights measured at additional routine check-ups. We included all children with at least two measurements of BMI in our analysis, who comprised 99% of the available sample. We parameterised the relationship between BMI *z*-score and age using linear splines and 3 knot points at 3, 12 and 34 months to describe periods of approximately linear growth based on the data [17]. Although a linear spline model is an approximation of the true growth function, its coefficients are easily interpretable and have been shown to produce good model fit in this and several other cohorts [30–35]. The multilevel model included three levels: (i) the age at measurement; (ii) the individual child; and (iii) the hospital or polyclinic site where the child was examined. The multilevel model allows completely unstructured covariances between phases of growth and allows negative correlations for adjacent growth phases. To predict the mean BMI *z*-scores in each category of maternal education at various ages, we employed the *lincom* command in STATA, which uses the multilevel model coefficients. To estimate absolute differences in BMI *z*-score between the highest and lowest categories of maternal education at age 7 years, we used precise model coefficients and the *lincom* command (see eMethods 1 for details). We also report observed measurements taken at planned clinic visits in terms of sex-specific WHO BMI-for-age *z*-scores [27, 28], thus allowing comparison with other cohorts.

### Crude and multivariable analyses of BMI *z*-score trajectories

Three multivariable multilevel models were fitted for BMI *z*-score per category of socioeconomic indicator (maternal education, paternal education and highest household occupation), which are the main exposures for this study. Model 1 controlled for study trial arm (breastfeeding promotion intervention or usual care [22]) and geographical location (urban versus rural and East versus West Belarus), as these geographical variables may confound the associations examined. Model 2 additionally controlled for parents’ BMIs, a proxy for adiposity-related genetic and environmental factors that may influence the child’s size [16]. Model 3 additionally controlled for maternal smoking status and number of older siblings, because previous studies have shown these factors to be associated with both childhood adiposity gain and socioeconomic position [18–21].

As we found strong evidence of a difference in the BMI *z*-score trajectories between girls and boys (*p* for sex interaction <0.001), we present results for girls and boys separately, although we observed little evidence that associations between socioeconomic indicators and BMI trajectories differed by sex (*p*-values for sex interaction for the main exposures ≥0.2; for maternal education *p* = 0.6; paternal education *p* = 0.2, and occupation *p* = 0.3). To verify the assumption of a linear association between categories of socioeconomic indicator and BMI *z*-score, we compared analyses using categories of socioeconomic indicators with analyses using their continuous forms, with likelihood ratio tests based on the fully-adjusted models. Evidence against the assumption of linearity was *p* ≥  0.03 (for maternal education these values were for girls *p* = 0.96 and for boys *p* = 0.03 and for paternal education, *p* = 0.05 and *p* = 0.45, respectively).

We modelled BMI *z*-score trajectories for 16,861 children with at least two measures of BMI (99% of the original cohort); we then restricted the analysis to those 12,385 children (73% of the original cohort) with complete data on all covariables. As findings did not differ substantively between these two data sets, we present adjusted results for those children with complete data only. Because most results were similar across socioeconomic indicators, we present those for maternal education in detail, with results for paternal education and highest household occupation as supplementary web tables. In a sensitivity analysis, we used the *ice* command in STATA to impute 20 values for each missing observation (including BMI *z*-scores at the scheduled clinic visits) for 16,861 individuals (see eMethods 2 for details). Analyses were conducted in STATA version 14.2 (StataCorp. 2013. Stata Statistical Software: Release 13. College Station, TX: StataCorp LP), using the *runmlwin* command [36] in MLwiN version 2.36 [37].

## Results

The 12,385 children with complete data who were included in our analyses had a total of 131,898 weight and length or height measurements (median: 11; IQR: 10–13; range: 3–14), including 37,888 measurements (median: 4; IQR: 4–5; range: 1–6) for 10,280 individuals abstracted from medical records between the 12-month and 6.5-year examination. Each category of parental education and highest household occupation had a similar number of measurements. Reasons for missing data were: 185 children with fewer than two measurements of BMI, 4414 individuals with one or both parental weight or height measurements missing and 62 parents with anthropometry measurements >±4 standard deviations from the mean. Comparisons of the 12,385 children with complete data to the 4476 with at least two measures of BMI but incomplete data (Web Table 1) revealed that children with incomplete data were more likely to have less educated mothers and were slightly shorter and lighter at birth.

**Fig. 1:**
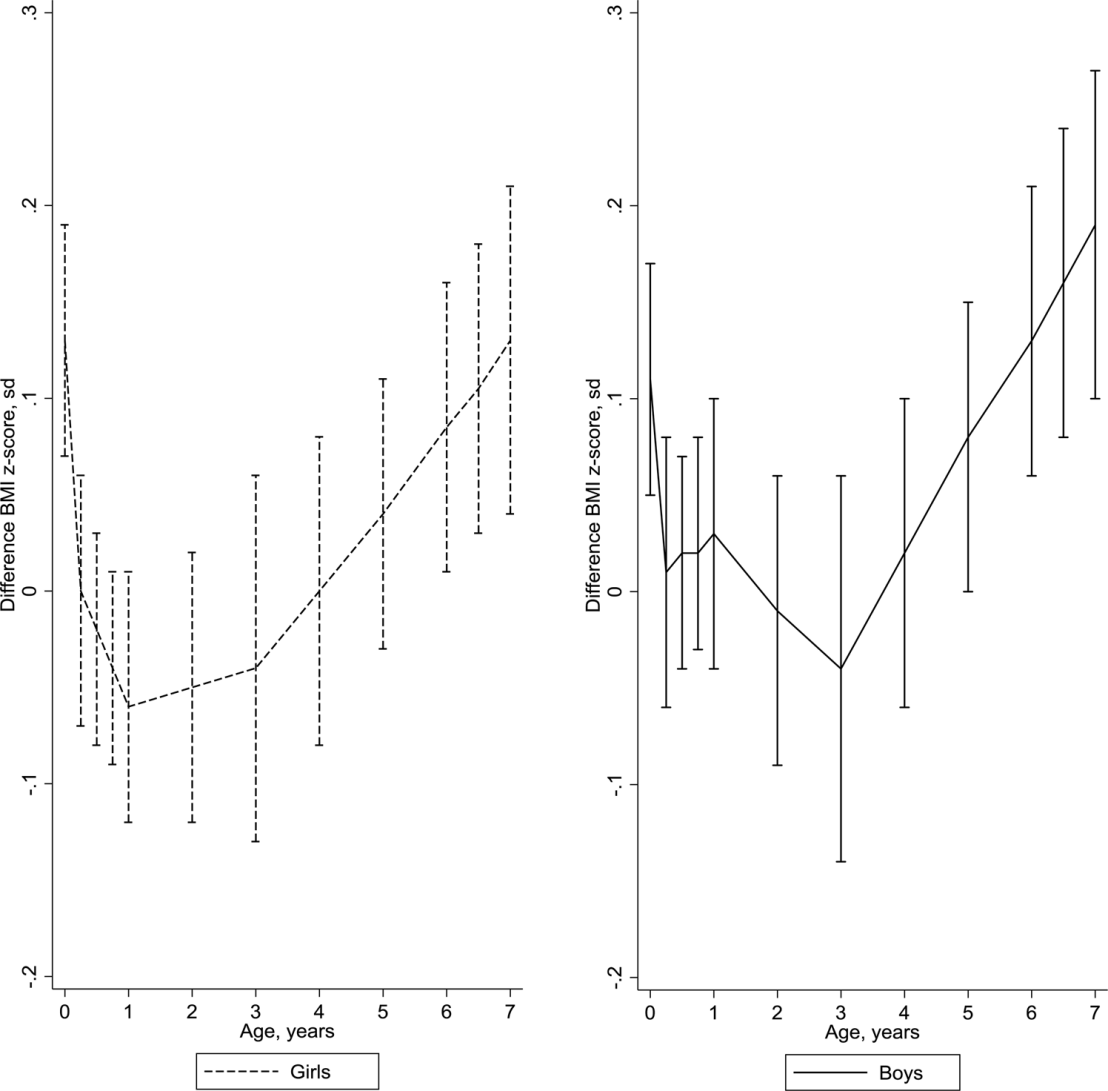
Absolute difference (and its 95% confidence interval) in BMI *z*-score between the highest versus lowest category of maternal education in girls and boys

Maternal educational attainment was positively associated with offspring BMI *z*-score at birth and after age 3 years for both girls and boys (Table 1). The mean difference in BMI *z*-score at birth between infants in the highest versus lowest categories of maternal education was 0.13 *z*-scores (95% CI: 0.07, 0.19) for girls and 0.11 *z*-scores (95% CI: 0.05, 0.17) for boys. In terms of weight for an infant of average birth length, these differences are equivalent to 43 and 38 g, respectively. After 3 years, absolute differences in BMI *z*-scores between the highest versus lowest categories of maternal education increased with age (Table 1; Fig. 1). Using the multilevel model coefficients, girls born to mothers with the highest educational attainment had mean *z*-scores 0.13 *z*-scores (95% CI: 0.04, 0.21) higher at age 7 years than those born to the least educated mothers; among boys, this difference was 0.19 *z*-scores (95% CI: 0.1, 0.27). These differences at age 7 years are equivalent to differences in BMI of 0.22 and 0.27 kg/m^2^, respectively, for a child of average height. Web Table 2 shows the observed mean weight-, length/height- and BMI-for-age *z*-scores at each of the planned clinic visits by categories of maternal education. BMI-for-age *z*-scores generally showed graded differences between socioeconomic groups at birth and 6.5 years.

**Table 1:** BMI *z*-score at various ages by category of maternal education, *N* = 16,861 (estimated from multilevel models)

	Predicted BMI *z*-score by category of maternal education (95% confidence interval)						Complete university compared to common or incomplete secondary	
Age	Initial, incomplete or common secondary		Advanced secondary or partial university		Completed university		Absolute difference in BMI *z*-scores	
Girls								
* N* = 8139	2943		4085		1111			
Birth	−0.64	(−0.7, −0.59)	−0.58	(−0.63, −0.53)	−0.51	(−0.58, −0.45)	0.13	(0.07, 0.19)
3 months	−0.08	(−0.14, −0.03)	−0.07	(−0.12, −0.02)	−0.09	(−0.16, −0.02)	0.00	(−0.07, 0.06)
6 months	0.37	(0.32, 0.42)	0.37	(0.32, 0.41)	0.35	(0.28, 0.41)	−0.02	(−0.08, 0.03)
9 months	0.82	(0.77, 0.87)	0.81	(0.76, 0.85)	0.78	(0.72, 0.84)	−0.04	(−0.09, 0.01)
1 year	1.28	(1.22, 1.33)	1.25	(1.2, 1.3)	1.22	(1.15, 1.29)	−0.06	(−0.12, 0.01)
2 years	0.78	(0.73, 0.84)	0.74	(0.69, 0.8)	0.73	(0.66, 0.8)	−0.05	(−0.12, 0.02)
3 years	0.36	(0.29, 0.43)	0.32	(0.26, 0.38)	0.32	(0.23, 0.41)	−0.04	(−0.13, 0.06)
4 years	0.23	(0.17, 0.29)	0.22	(0.16, 0.27)	0.23	(0.15, 0.31)	0.00	(−0.08, 0.08)
5 years	0.09	(0.04, 0.15)	0.12	(0.06, 0.17)	0.14	(0.07, 0.21)	0.04	(−0.03, 0.11)
6 years	−0.04	(−0.1, 0.02)	0.01	(−0.04, 0.07)	0.05	(−0.03, 0.12)	0.08	(0.01, 0.16)
6.5 years	−0.11	(−0.16, −0.05)	−0.04	(−0.09, 0.02)	0.00	(−0.08, 0.08)	0.11	(0.03, 0.18)
7 years	−0.17	(−0.23, −0.11)	−0.09	(−0.14, −0.03)	−0.05	(−0.13, 0.04)	0.13	(0.04, 0.21)
Boys								
* N* = 8722	3143		4395		1184			
Birth	−0.61	(−0.67, −0.56)	−0.53	(−0.58, −0.47)	−0.50	(−0.57, −0.43)	0.11	(0.05, 0.17)
3 months	−0.23	(−0.29, −0.17)	−0.16	(−0.22, −0.1)	−0.22	(−0.3, −0.15)	0.01	(−0.06, 0.08)
6 months	0.26	(0.21, 0.32)	0.32	(0.26, 0.37)	0.28	(0.21, 0.35)	0.02	(−0.04, 0.07)
9 months	0.76	(0.7, 0.82)	0.79	(0.74, 0.85)	0.78	(0.72, 0.85)	0.02	(−0.03, 0.08)
1 year	1.26	(1.2, 1.32)	1.27	(1.21, 1.32)	1.29	(1.21, 1.36)	0.03	(−0.04, 0.1)
2 years	0.79	(0.73, 0.85)	0.78	(0.72, 0.84)	0.77	(0.7, 0.85)	−0.01	(−0.09, 0.06)
3 years	0.39	(0.32, 0.47)	0.38	(0.31, 0.44)	0.35	(0.26, 0.45)	−0.04	(−0.14, 0.06)
4 years	0.29	(0.22, 0.36)	0.31	(0.25, 0.37)	0.31	(0.22, 0.39)	0.02	(−0.06, 0.1)
5 years	0.19	(0.12, 0.25)	0.23	(0.18, 0.29)	0.26	(0.18, 0.34)	0.08	(0, 0.15)
6 years	0.08	(0.02, 0.15)	0.16	(0.11, 0.22)	0.22	(0.14, 0.29)	0.13	(0.06, 0.21)
6.5 years	0.03	(−0.03, 0.1)	0.13	(0.07, 0.19)	0.19	(0.11, 0.28)	0.16	(0.08, 0.24)
7 years	−0.02	(−0.09, 0.05)	0.09	(0.03, 0.15)	0.17	(0.08, 0.26)	0.19	(0.1, 0.27)

Table 2 shows the BMI *z*-score change per increase in category of maternal education. In model 1, children of the most educated mothers had a lower BMI trajectory than children of the least educated mothers up to age 1 year. The greatest differences between socioeconomic groups were observed at ages 34 months to 7 years: 0.02 higher BMI *z*-scores per category of maternal education (95% CI: 0, 0.03; *p* for trend = 0.01) among girls and 0.03 *z*-scores (95% CI: 0.01, 0.04; *p* for trend < 0.001) among boys. Overall, children of the most educated mothers had a higher BMI trajectory than children of the least educated mothers after age 3 years. Findings were similar for children with (Table 2), and without complete data (Web Table 3).

**Table 2:** Mean difference in BMI-for-age *z*-score per increase in category of maternal education, *N* = 12,385 (estimated from multilevel models)

	Mean difference in BMI-for-age *z*-score per category of mother’s education								
	Uncontrolled		Model 1		Model 2		Model 3		
	Coef^a^ (95% CI^b^)	*p* for trend^c^	Coef (95% CI)	*p* for trend^c^	Coef (95% CI)	*p* for trend^c^	Coef (95% CI)		*p* for trend^c^
Girls, *N* = 5969									
BMI *z*-score at birth	0.05 (0.01, 0.08)	0.005	0.04 (0.01, 0.08)	0.02	0.04 (0, 0.07)	0.03	0.03 (−0.01, 0.06)		0.14
BMI *z*-score trajectory from:									
0–3 months	−0.19 (−0.36, −0.01)	0.04	−0.15 (−0.32, 0.02)	0.09	−0.16 (−0.33, 0.02)	0.08	−0.11 (−0.28, 0.07)		0.22
>3–12 months	−0.05 (−0.11, 0)	0.05	−0.04 (−0.09, 0.02)	0.21	−0.04 (−0.09, 0.02)	0.20	−0.03 (−0.09, 0.03)		0.29
>12–34 months	0.00 (−0.03, 0.03)	0.81	0.00 (−0.03, 0.03)	0.93	0.00 (−0.03, 0.03)	0.95	0.00 (−0.03, 0.03)		0.87
>34–84 months	0.02 (0.01, 0.04)	0.001	0.02 (0, 0.03)	0.01	0.02 (0, 0.03)	0.03	0.02 (0, 0.03)		0.02
Boys, *N* = 6416									
BMI *z*-score at birth	0.06 (0.03, 0.1)	<0.001	0.05 (0.02, 0.09)	0.002	0.05 (0.02, 0.09)	0.002	0.05 (0.01, 0.08)		0.009
BMI *z*-score trajectory from									
0–3 months	−0.19 (−0.37, −0.01)	0.04	−0.15 (−0.33, 0.03)	0.09	−0.17 (−0.35, 0.01)	0.06	−0.15 (−0.33, 0.03)		0.11
>3–12 months	−0.01 (−0.07, 0.05)	0.67	0.00 (−0.05, 0.06)	0.88	0.01 (−0.05, 0.07)	0.83	0.01 (−0.05, 0.07)		0.64
>12–34 months	−0.02 (−0.05, 0.01)	0.25	−0.02 (−0.05, 0.01)	0.14	−0.02 (−0.05, 0.01)	0.13	−0.02 (−0.06, 0.01)		0.13
>34–84 months	0.03 (0.02, 0.05)	<0.001	0.03 (0.01, 0.04)	<0.001	0.03 (0.01, 0.04)	0.001	0.02 (0.01, 0.04)		0.002

Model 1: controlled for study trial arm, urban or rural location and East or West of Belarus

Model 2: as model 1 additionally controlled for both parents’ BMI

Model 3: as model 2 additionally controlled for maternal smoking (never, ever or unknown) and older siblings (none, 1 or >1)

^a^ Coef = coefficient

^b^ CI = confidence interval

^c^ Trend across exposure categories

Using the precise model coefficients, we predicted absolute differences in *z*-score in the highest versus lowest categories of education. After adjusting for trial arm and geographical location (model 1), children born to mothers in the highest versus lowest categories had higher mean BMI *z*-scores at age 7 by 0.11 *z*-scores (95% CI: 0.03, 0.19) among girls and 0.18 *z*-scores (95% CI: 0.1, 0.27) among boys. These differences at age 7 years are equivalent to differences in BMI of 0.19 and 0.26 kg/m^2^, respectively, for a child of average height at 7 years. After adjusting for parental BMI (model 2), differences in mean BMI *z*-score at 7 years attenuated to 0.08 *z*-scores (95% CI: 0, 0.16) among girls and 0.16 *z*-scores (95% CI: 0.07, 0.24) among boys. Little difference was seen between adjusting for mother’s or father’s BMI separately (Web Table 4). Further controlling for maternal smoking and number of older siblings (Table 2, model 3) also made little additional difference to the association between maternal education and BMI *z*-score trajectory after infancy [0.09 (95% CI: 0, 0.17) among girls and 0.15 (95% CI: 0.07, 0.23) among boys].

Associations of paternal education (Web Table 5) and highest household occupation (Web Table 6) with offspring BMI trajectories were similar to those observed for maternal education. In fully controlled models, girls at age 7 years in the highest versus lowest paternal education categories had BMI *z*-scores that were 0.05 *z*-scores (95% CI: −0.03, 0.13) higher, and those from non-manual versus manual household occupations were 0.08 *z*-scores (95% CI: 0.02, 0.13) higher. The corresponding differences for boys were 0.11 *z*-scores (95% CI: 0.02, 0.19) and 0.13 *z*-scores (95% CI: 0.08, 0.19), respectively. Multiple imputation of missing data in Web Tables 7–9 did not alter these conclusions.

## Discussion

In this study of over 12,000 healthy children born at term in the Republic of Belarus, those born to the most educated mothers had BMI *z*-scores approximately 0.12 *z*-scores (equivalent to 38–43 g for a child with average birth length) heavier at birth than those born to the least educated mothers. BMI trajectories from ~3 to 7 years differed among socioeconomic groups; children of the most educated mothers had higher trajectories than those of the least educated mothers. Further adjusting for parental BMI explained only a part of these early-life differences. Findings were similar when we examined paternal education or highest household occupation as the exposures of interest.

Systematic reviews have shown that in low- and middle-income countries, children from more affluent or educated families have higher levels of childhood obesity [5]. This is consistent with our previous examination of associations of socioeconomic position with overweight and obesity in Belarus when the children were aged 6.5 years [15], and with our current study of BMI trajectories. Parental BMI explained little of these socioeconomic differences, although parental overweight has been shown to influence offspring adiposity in our studies and others [38–40].

We found that BMI differences by maternal educational attainment increased substantially after age 3 years. In Belarus at the time of recruitment for PROBIT, maternity leave was an obligatory 3 years [22]. By age 3, weaning has already occurred and children are more mobile. The socioeconomic differences we observed suggest that environmental factors, such as family diet and physical activity and factors related to child care and schools, may more strongly influence offspring BMI after that age, unfortunately, these factors were not measured in the PROBIT cohort. Although Belarus has relatively low levels of childhood overweight and obesity (~10% in Belarus compared to ~23% in developed countries) [15, 41], the downstream consequences of excess adiposity are a major public health concern and include increases in morbidity and mortality rates and health care needs.

Although *z*-scores are widely used to track adiposity with age, they have limitations. Most importantly, BMI is a measure of weight for length or height—not a measure of adiposity. Moreover, if *z*-scores are internally standardised, findings may not be generalisable to other populations; or, as in our study, if externally standardised, the entire sample would be compared to a reference population, such as that of the USA, which may have very different overall prevalence of overweight and obesity and not necessarily exhibit optimal growth. Absolute BMI could be modelled although BMI curves are complex and many data points around the suggested knots may be necessary to model them accurately.

Our results are similar to those from other middle-income countries. A small study of 255 children aged 0.1–5.5 years followed for 4.9–7.5 years from a poor area of Brazil observed that children from families with household assets above the cohort median had greater BMI-for-age *z*-scores after 7 years than those below the median [42]. Another study in Brazil of 1458 children measured at birth and 9–15 months later observed that children from the highest-income families were on average heavier at birth and gained 20% more weight (independent of birth weight) than those from the lowest-income families [8]. A study in Russia found no association between maternal education and occupation and weight-for-length *z*-scores, based on linear regression of *z*-scores at birth and 12 months [9]. That study combined data from 1067 girls and boys and recorded only two measurements of weight and length. In contrast, we observed that socioeconomic differences were already apparent at birth. In high-income settings, less educated socioeconomic groups generally experience greater obesity [6]. For example, socioeconomic differences in BMI trajectories emerged by age 4 years in a UK cohort, with lower mean BMI among children of university-educated mothers (as opposed to higher BMI found in our study) [30].

### Strengths and limitations

Strengths of our study include its large sample size, prospective measurements of weight, length and height over a long period, and a modelling strategy that was not restricted to individuals with complete data at all time-points or data measured at exactly the same age for all individuals. This novel method for examining BMI *z*-score trajectories overcomes many of the limitations of other more conventional methods, as mentioned previously. One limitation of our study is its basis on BMI, which does not measure fatness per se; for example, if a child is very muscular, his or her weight might be high for height and age but would not represent excess fat [43]; however, this is unlikely in this age group. Another limitation is that measurements in infancy and childhood were based on routine child health records (only measurements at the 6.5 year follow-up were standardised and audited). We were therefore unable to assess the reliability of the routinely-collected weight, length and height measurements during infancy; associations may therefore have been attenuated by non-differential measurement error. Children without complete data were more likely to have less educated mothers and were slightly shorter and lighter at birth. However, those differences should not affect the associations we observed between family socioeconomic position and postnatal BMI trajectories. Finally, parental education and household occupation were examined as static exposures. Although these socioeconomic measures were unlikely to have changed over the period of observation, we were unable to examine the relationship between BMI trajectories and dynamically changing measures of socioeconomic position, such as instability in income or food security.

## Conclusions

In summary, despite low reported levels of income inequality in this population in Belarus [13], our findings suggest that socioeconomic differences in BMI trajectories are present at birth and then increase after 3 years of age, with children from more educated or non-manual households showing higher trajectories than those from less educated or manual households, in contrast to findings in other settings [4, 6]. Relative to other countries, Belarus has low levels of overweight and obesity.

## Disclaimer

The views expressed are those of the authors and not necessarily of any funding body.

## Electronic supplementary material

Supplementary Web Materials
