# Supplementary material for: Socioeconomic differences in childhood BMI trajectories in Belarus
Source: Int J Obes (Lond). 2018 Feb 28;42(9):1651–60. doi: 10.1038/s41366-018-0042-0 (PMC6033313; doi:10.1038/s41366-018-0042-0)
Supplement: Supplementary file 1 — Supplementary Web Materials [file 41366_2018_42_MOESM1_ESM.docx]

# **Web Table 1 Characteristics of 12,385 children with complete data on all covariables versus 4,476 children with at least two measurements of BMI but with incomplete data on all covariables**

|  | **Girls** | | | | | | | |  | **Boys** | | | | | | | |
| --- | --- | --- | --- | --- | --- | --- | --- | --- | --- | --- | --- | --- | --- | --- | --- | --- | --- |
|  | **Those with complete data (5,969)** | | |  | **Those with incomplete data (2,170)** | | | **P for**  **diff ^a^** |  | **Those with complete data (6,416)** | | |  | **Those with incomplete data (2,306)** | | | **P for**  **diff^a^** |
|  | **n** | **Mean** | **SD** |  | **n** | **Mean** | **SD** |  |  | **n** | **Mean** | **SD** |  | **n** | **Mean** | **SD** |  |
| Birth length, cm | 5,969 | 51.7 | 2.0 |  | 2,170 | 51.5 | 2.0 | <0.001 |  | 6,416 | 52.3 | 2.2 |  | 2,306 | 52.1 | 2.2 | <0.001 |
| Birth weight, g | 5,969 | 3376 | 404.2 |  | 2,170 | 3340 | 385 | <0.001 |  | 6,416 | 3512 | 423.1 |  | 2,306 | 3483.9 | 435.1 | 0.007 |
| Maternal BMI | 5,969 | 24.5 | 4.3 |  | 635 | 23.8 | 3.9 | <0.001 |  | 6,416 | 24.5 | 4.3 |  | 644 | 23.9 | 4.0 | <0.001 |
| Paternal BMI | 5,969 | 25.6 | 3.2 |  | 36 | 25.9 | 4.1 | 0.6 |  | 6,416 | 25.7 | 3.2 |  | 36 | 26.1 | 3.9 | 0.5 |
|  | **n** | **%** |  |  | **n** | **%** |  |  |  | **n** | **%** |  |  | **n** | **%** |  |  |
| Maternal education |  |  |  |  |  |  |  |  |  |  |  |  |  |  |  |  |  |
| Initial, incomplete or common secondary | 2,064 | 35 |  |  | 879 | 41 |  |  |  | 2,238 | 35 |  |  | 905 | 39 |  |  |
| Advance secondary or part university | 3,076 | 52 |  |  | 1,009 | 47 |  |  |  | 3,301 | 51 |  |  | 1094 | 47 |  |  |
| Completed university | 829 | 14 |  |  | 282 | 13 |  | <0.001 |  | 877 | 14 |  |  | 307 | 13 |  | 0.001 |
| Urban location (versus rural) | 3,258 | 55 |  |  | 1,457 | 67 |  | <0.001 |  | 3,543 | 55 |  |  | 1,629 | 71 |  | <0.001 |
| West Belarus (versus East) | 3,132 | 52 |  |  | 1,003 | 46 |  | <0.001 |  | 3,465 | 54 |  |  | 1,038 | 45 |  | <0.001 |
| In breastfeeding promotion arm | 3,005 | 50 |  |  | 1,231 | 57 |  | <0.001 |  | 3,201 | 50 |  |  | 1,316 | 57 |  | <0.001 |
| Maternal smoking |  |  |  |  |  |  |  |  |  |  |  |  |  |  |  |  |  |
| Unknown | 238 | 4 |  |  | 1,444 | 67 |  |  |  | 273 | 4 |  |  | 1,534 | 67 |  |  |
| Ever | 740 | 12 |  |  | 277 | 13 |  |  |  | 782 | 12 |  |  | 307 | 13 |  |  |
| Never | 4,991 | 84 |  |  | 449 | 21 |  | <0.001 |  | 5,361 | 84 |  |  | 465 | 20 |  | <0.001 |
| Older siblings |  |  |  |  |  |  |  |  |  |  |  |  |  |  |  |  |  |
| None | 3,280 | 55 |  |  | 1,414 | 65 |  |  |  | 3,558 | 55 |  |  | 1,501 | 65 |  |  |
| 1 | 2,174 | 36 |  |  | 587 | 27 |  |  |  | 2,303 | 36 |  |  | 609 | 26 |  |  |
| 2 or more | 515 | 9 |  |  | 169 | 8 |  | <0.001 |  | 555 | 9 |  |  | 195 | 8 |  | <0.001 |

^a^ p for difference between those with complete data to those with incomplete data, for those with incomplete data any data available is compared (ttest and chi squared test)

# **Web Table 2 Observed mean and standard deviation weight (kg), length or height (cm) and BMI-for-age z-scores^a^ at clinic visits, by categories of maternal education**

|  | **Initial,** **incomplete or common secondary** | | | | |  | **Advanced secondary or partial university** | | | | |  | **Completed university** | | | | |
| --- | --- | --- | --- | --- | --- | --- | --- | --- | --- | --- | --- | --- | --- | --- | --- | --- | --- |
| **Clinic visits** | **N** | **Weight** | **Length or height** | **BMI-for-age z-score** |  |  | **N** | **Weight** | **Length or height** | **BMI-for-age z-score** |  |  | **N** | **Weight** | **Length or height** | **BMI-for-age z-score** |  |
| **Girls** |  |  |  |  |  |  |  |  |  |  |  |  |  |  |  |  |  |
| **Birth** | 2,943 | 3.3 (0.4) | 51.4 (2.0) | -0.69 (0.97) |  |  | 4,085 | 3.4 (0.4) | 51.7 (2.0) | -0.62 (0.94) |  |  | 1,111 | 3.4 (0.4) | 51.9 (2.0) | -0.56 (0.93) |  |
| **1m** | 2,845 | 4.2 (0.5) | 54.1 (2.0) | -0.38 (0.89) |  |  | 3,912 | 4.2 (0.5) | 54.5 (2.0) | -0.32 (0.89) |  |  | 1,053 | 4.3 (0.5) | 54.6 (1.9) | -0.3 (0.89) |  |
| **2m** | 2,786 | 5.0 (0.5) | 57.0 (2.1) | -0.33 (0.92) |  |  | 3,868 | 5.1 (0.5) | 57.4 (2.1) | -0.29 (0.9) |  |  | 1,045 | 5.1 (0.5) | 57.5 (2.0) | -0.27 (0.89) |  |
| **3m** | 2,838 | 5.8 (0.6) | 60.1 (2.3) | -0.17 (0.95) |  |  | 3,958 | 5.9 (0.6) | 60.5 (2.2) | -0.15 (0.93) |  |  | 1,072 | 6.0 (0.6) | 60.6 (2.3) | -0.15 (0.93) |  |
| **6m** | 2,808 | 7.8 (0.8) | 66.1 (2.6) | 0.49 (1.01) |  |  | 3,947 | 7.9 (0.8) | 66.7 (2.5) | 0.49 (0.97) |  |  | 1,071 | 7.9 (0.8) | 66.8 (2.4) | 0.46 (0.92) |  |
| **9m** | 2,702 | 9.1 (0.9) | 70.8 (2.7) | 0.91 (0.92) |  |  | 3,791 | 9.3 (0.9) | 71.3 (2.5) | 0.91 (0.91) |  |  | 1,026 | 9.3 (0.9) | 71.4 (2.4) | 0.87 (0.86) |  |
| **12m** | 2,476 | 10.2 (0.9) | 75.0 (2.7) | 1.15 (0.87) |  |  | 3,484 | 10.4 (0.9) | 75.5 (2.5) | 1.14 (0.87) |  |  | 959 | 10.4 (1.0) | 75.6 (2.5) | 1.14 (0.86) |  |
| **…** |  |  |  |  |  |  |  |  |  |  |  |  |  |  |  |  |  |
| **6.5y** | 2,184 | 22.0 (3.5) | 119.5 (5.1) | -0.11 (0.97) |  |  | 3,185 | 22.5 (3.6) | 120.4 (5.0) | -0.02 (0.98) |  |  | 820 | 23.0 (3.8) | 121.5 (5.2) | 0 (0.98) |  |
|  |  |  |  |  |  |  |  |  |  |  |  |  |  |  |  |  |  |
| **Boys** |  |  |  |  |  |  |  |  |  |  |  |  |  |  |  |  |  |
| **Birth** | 3,143 | 3.5 (0.4) | 52.1 (2.2) | -0.59 (0.94) |  |  | 4,395 | 3.5 (0.4) | 52.3 (2.2) | -0.51 (0.92) |  |  | 1,184 | 3.5 (0.4) | 52.4 (2.1) | -0.46 (0.91) |  |
| **1m** | 3,019 | 4.4 (0.5) | 55.0 (2.1) | -0.49 (1) |  |  | 4,214 | 4.5 (0.5) | 55.2 (2.1) | -0.37 (0.97) |  |  | 1,128 | 4.5 (0.5) | 55.3 (2.1) | -0.42 (0.97) |  |
| **2m** | 2,969 | 5.3 (0.6) | 58.1 (2.3) | -0.48 (1.01) |  |  | 4,186 | 5.4 (0.6) | 58.4 (2.3) | -0.36 (0.99) |  |  | 1,124 | 5.4 (0.6) | 58.5 (2.2) | -0.39 (1.01) |  |
| **3m** | 3,039 | 6.2 (0.7) | 61.3 (2.5) | -0.27 (1.02) |  |  | 4,273 | 6.4 (0.7) | 61.7 (2.4) | -0.21 (1.02) |  |  | 1,148 | 6.4 (0.7) | 61.9 (2.3) | -0.25 (1.02) |  |
| **6m** | 3,008 | 8.2 (0.8) | 67.5 (2.7) | 0.46 (1.07) |  |  | 4,243 | 8.4 (0.8) | 68.0 (2.6) | 0.48 (1.06) |  |  | 1,135 | 8.4 (0.8) | 68.3 (2.5) | 0.45 (0.98) |  |
| **9m** | 2,904 | 9.6 (0.9) | 72.2 (2.7) | 0.87 (0.98) |  |  | 4,100 | 9.8 (0.9) | 72.6 (2.5) | 0.91 (0.99) |  |  | 1,090 | 9.8 (0.9) | 72.9 (2.5) | 0.9 (0.93) |  |
| **12m** | 2,677 | 10.7 (1.0) | 76.2 (2.7) | 1.11 (0.94) |  |  | 3,754 | 10.9 (1.0) | 76.6 (2.5) | 1.16 (0.95) |  |  | 1,050 | 10.9 (0.9) | 76.8 (2.6) | 1.16 (0.9) |  |
| **…** |  |  |  |  |  |  |  |  |  |  |  |  |  |  |  |  |  |
| **6.5y** | 2,315 | 22.4 (3.2) | 119.8 (4.9) | 0.02 (0.98) |  |  | 3,401 | 23.1 (3.5) | 120.8 (5.1) | 0.14 (1.03) |  |  | 885 | 23.6 (3.7) | 121.6 (4.7) | 0.2 (1.15) |  |

^a^ WHO Child Growth Standards 2006 (0-5 years)[^26^](#_ENREF_26)/WHO Reference 2007 (5-19 years)[^27^](#_ENREF_27), using the STATA command *zanthro*^[25](#_ENREF_25)^.

# **Web Table 3 BMI-for-age z-score by category of maternal education N=16,861 (estimated from multilevel models)**

|  | **Mean BMI-for-age z-score by category of maternal education, kg/year (95% confidence interval)** | | | | | |  | **Mean difference in BMI-for-age z-score per category of maternal education**  **^a^ Coef (95% ^b^ CI)** | | **p for trend** ^a^ |
| --- | --- | --- | --- | --- | --- | --- | --- | --- | --- | --- |
|  | **Initial,** **incomplete or common secondary** | | **Advance secondary or partial university** | | **Completed university** | | **p for difference between groups** |  |  |  |
| **Girls** |  |  |  |  |  |  |  |  |  |  |
| **N=8,139** | **2,943** |  | **4,085** |  | **1,111** |  |  |  |  |  |
| BMI z-score at birth | -0.64 | (-0.7, -0.59) | -0.58 | (-0.63, -0.53) | -0.51 | (-0.58, -0.45) | <0.001 | 0.06 | (0.03, 0.09) | <0.001 |
| BMI z-score trajectory from:  0 - 3 months | 2.23 | (2.07, 2.4) | 2.01 | (1.87, 2.15) | 1.70 | (1.44, 1.97) | 0.003 | -0.25 | (-0.4, -0.1) | 0.001 |
| >3 - 12 months | 1.81 | (1.76, 1.87) | 1.76 | (1.72, 1.81) | 1.74 | (1.65, 1.83) | 0.23 | -0.04 | (-0.09, 0.01) | 0.10 |
| >12 - 34 months | -0.49 | (-0.52, -0.46) | -0.50 | (-0.53, -0.48) | -0.49 | (-0.54, -0.44) | 0.80 | 0.00 | (-0.03, 0.03) | 0.99 |
| >34 - 84 months | -0.13 | (-0.15, -0.12) | -0.10 | (-0.11, -0.09) | -0.09 | (-0.12, -0.07) | 0.002 | 0.02 | (0.01, 0.04) | 0.001 |
|  |  |  |  |  |  |  |  |  |  |  |
| **Boys** |  |  |  |  |  |  |  |  |  |  |
| **N=8,722** | **3,143** |  | **4,395** |  | **1,184** |  |  |  |  |  |
| BMI z-score at birth | -0.61 | (-0.67, -0.56) | -0.53 | (-0.58, -0.47) | -0.50 | (-0.57, -0.43) | <0.001 | 0.06 | (0.04, 0.09) | <0.001 |
| BMI z-score trajectory from:  0 - 3 months | 1.53 | (1.36, 1.7) | 1.47 | (1.32, 1.61) | 1.12 | (0.84, 1.39) | 0.04 | -0.17 | (-0.32, -0.02) | 0.03 |
| >3 - 12 months | 1.99 | (1.93, 2.05) | 1.90 | (1.85, 1.95) | 2.02 | (1.92, 2.11) | 0.02 | -0.01 | (-0.06, 0.04) | 0.66 |
| >12 - 34 months | -0.47 | (-0.5, -0.44) | -0.49 | (-0.51, -0.46) | -0.51 | (-0.57, -0.46) | 0.35 | -0.02 | (-0.05, 0.01) | 0.16 |
| >34 - 84 months | -0.10 | (-0.12, -0.09) | -0.07 | (-0.08, -0.06) | -0.05 | (-0.07, -0.02) | <0.001 | 0.03 | (0.02, 0.04) | <0.001 |

^a^ Trend across exposure categories

# **Web Table 4 Mean difference in BMI-for-age z-score per increase in category of maternal education, N=12,385 (estimated from multilevel models)**

|  | **Mean difference in BMI-for-age z-score per category of maternal education** | | | | | | | | | | | | | | |
| --- | --- | --- | --- | --- | --- | --- | --- | --- | --- | --- | --- | --- | --- | --- | --- |
|  | **Model 1** | | |  | **Model 1 +**  **maternal BMI** | | |  | **Model 1 +**  **paternal BMI** | | |  | **Model 1+**  **maternal and paternal BMI** | | |
|  | ^a^ **Coef (95%^b^ CI)** | | **P for trend** ^c^ |  | **Coef (95% CI)** | | **P for trend** ^c^ |  | **Coef (95% CI)** | | **P for trend** ^c^ |  | **Coef (95% CI)** | | **P for trend** ^c^ |
| **Girls N=5,969** |  |  |  |  |  |  |  |  |  |  |  |  |  |  |  |
| BMI z-score at birth | 0.04 | (0.01, 0.08) | 0.02 |  | 0.04 | (0.01, 0.08) | 0.02 |  | 0.03 | (0, 0.07) | 0.06 |  | 0.04 | (0, 0.07) | 0.03 |
| BMI z-score trajectory from:  0 - 3 months | -0.15 | (-0.32, 0.02) | 0.09 |  | -0.15 | (-0.33, 0.02) | 0.08 |  | -0.14 | (-0.32, 0.03) | 0.10 |  | -0.16 | (-0.33, 0.02) | 0.08 |
| >3 - 12 months | -0.04 | (-0.09, 0.02) | 0.21 |  | -0.04 | (-0.09, 0.02) | 0.21 |  | -0.04 | (-0.09, 0.02) | 0.19 |  | -0.04 | (-0.09, 0.02) | 0.20 |
| >12 - 34 months | 0.00 | (-0.03, 0.03) | 0.93 |  | 0.00 | (-0.03, 0.03) | 0.94 |  | 0.00 | (-0.03, 0.03) | 0.92 |  | 0.00 | (-0.03, 0.03) | 0.95 |
| >34 - 84 months | 0.02 | (0, 0.03) | 0.009 |  | 0.02 | (0.01, 0.03) | 0.007 |  | 0.02 | (0, 0.03) | 0.04 |  | 0.02 | (0, 0.03) | 0.03 |
|  |  |  |  |  |  |  |  |  |  |  |  |  |  |  |  |
| **Boys N=6,416** |  |  |  |  |  |  |  |  |  |  |  |  |  |  |  |
| BMI z-score at birth | 0.05 | (0.02, 0.09) | 0.002 |  | 0.06 | (0.02, 0.09) | 0.001 |  | 0.05 | (0.02, 0.08) | 0.004 |  | 0.05 | (0.02, 0.09) | 0.002 |
| BMI z-score trajectory from:  0 - 3 months | -0.15 | (-0.33, 0.03) | 0.09 |  | -0.16 | (-0.34, 0.02) | 0.079 |  | -0.16 | (-0.34, 0.02) | 0.08 |  | -0.17 | (-0.35, 0.01) | 0.06 |
| >3 - 12 months | 0.00 | (-0.05, 0.06) | 0.88 |  | 0.01 | (-0.05, 0.07) | 0.85 |  | 0.00 | (-0.06, 0.06) | 0.88 |  | 0.01 | (-0.05, 0.07) | 0.83 |
| >12 - 34 months | -0.02 | (-0.05, 0.01) | 0.14 |  | -0.02 | (-0.05, 0.01) | 0.15 |  | -0.02 | (-0.05, 0.01) | 0.13 |  | -0.02 | (-0.05, 0.01) | 0.13 |
| >34 - 84 months | 0.03 | (0.01, 0.04) | <0.001 |  | 0.03 | (0.01, 0.04) | <0.001 |  | 0.02 | (0.01, 0.04) | 0.001 |  | 0.03 | (0.01, 0.04) | 0.001 |

Model 1: length or height, study trial arm, urban or rural location and East or West of Belarus

^a^ Coef=coefficient

^b^ CI=confidence interval

^c^ Trend across exposure categories

# **Web Table 5 Mean BMI z-score per one unit increase in category of paternal education, N=12,126 (estimated from multilevel models)**

|  | **Difference in BMI z-score per category of paternal education** | | | | | | | | | | | | | | |
| --- | --- | --- | --- | --- | --- | --- | --- | --- | --- | --- | --- | --- | --- | --- | --- |
|  | **Uncontrolled** | | |  | **Model 1** | | |  | **Model 2** | | |  | **Model 3** | | |
|  | ^a^ **Coef**  **(95%^b^ CI)** | | **P for trend** ^c^ |  | **Coef**  **(95% CI)** | | **P for trend** ^c^ |  | **Coef**  **(95% CI)** | | **P for trend** ^c^ |  | **Coef**  **(95% CI)** | | **P for trend** ^c^ |
| **Girls N=5,843** |  |  |  |  |  |  |  |  |  |  |  |  |  |  |  |
| BMI z-score at birth | 0.05 | (0.01, 0.08) | 0.006 |  | 0.04 | (0.01, 0.08) | 0.02 |  | 0.04 | (0.01, 0.08) | 0.01 |  | 0.03 | (0, 0.07) | 0.06 |
| BMI z-score trajectory from:  0 - 3 months | -0.20 | (-0.37, -0.03) | 0.02 |  | -0.18 | (-0.35, 0) | 0.04 |  | -0.20 | (-0.37, -0.02) | 0.03 |  | -0.16 | (-0.33, 0.02) | 0.08 |
| >3 - 12 months | -0.02 | (-0.08, 0.03) | 0.46 |  | 0.00 | (-0.06, 0.05) | 0.98 |  | 0.00 | (-0.06, 0.06) | 1.0 |  | 0.01 | (-0.05, 0.06) | 0.80 |
| >12 - 34 months | 0.01 | (-0.02, 0.04) | 0.65 |  | 0.00 | (-0.03, 0.03) | 0.85 |  | 0.00 | (-0.03, 0.03) | 0.78 |  | 0.00 | (-0.03, 0.03) | 0.88 |
| >34 - 84 months | 0.01 | (0, 0.03) | 0.11 |  | 0.01 | (-0.01, 0.02) | 0.40 |  | 0.00 | (-0.01, 0.02) | 0.50 |  | 0.00 | (-0.01, 0.02) | 0.50 |
|  |  |  |  |  |  |  |  |  |  |  |  |  |  |  |  |
| **Boys N=6,283** |  |  |  |  |  |  |  |  |  |  |  |  |  |  |  |
| BMI z-score at birth | 0.04 | (0, 0.07) | 0.04 |  | 0.03 | (0, 0.06) | 0.08 |  | 0.04 | (0, 0.07) | 0.03 |  | 0.03 | (0, 0.06) | 0.09 |
| BMI z-score trajectory from:  0 - 3 months | -0.04 | (-0.22, 0.14) | 0.65 |  | -0.02 | (-0.2, 0.15) | 0.79 |  | -0.06 | (-0.24, 0.12) | 0.51 |  | -0.03 | (-0.21, 0.14) | 0.72 |
| >3 - 12 months | -0.04 | (-0.1, 0.02) | 0.15 |  | -0.03 | (-0.09, 0.03) | 0.37 |  | -0.02 | (-0.08, 0.04) | 0.48 |  | -0.02 | (-0.07, 0.04) | 0.60 |
| >12 - 34 months | -0.03 | (-0.06, 0) | 0.05 |  | -0.03 | (-0.06, 0) | 0.03 |  | -0.03 | (-0.06, 0) | 0.04 |  | -0.03 | (-0.06, 0) | 0.04 |
| >34 - 84 months | 0.03 | (0.01, 0.04) | <0.001 |  | 0.03 | (0.01, 0.04) | 0.001 |  | 0.03 | (0.01, 0.04) | 0.001 |  | 0.02 | (0.01, 0.04) | 0.001 |

Model 1: controlled for length or height, study trial arm, urban or rural location and East or West of Belarus

Model 2: as Model 1 additionally controlled for parents’ BMI

Model 3: as Model 2 additionally controlled for maternal smoking (never, ever or unknown) and older siblings (none, 1 or >1)

^a^ Coef=coefficient

^b^ CI=confidence interval

^c^ Trend across exposure categories

# **Web Table 6 Mean BMI z -score per one unit increase in category of highest household occupation, N=11,690 (estimated from multilevel models)**

|  | **Difference in BMI z-score, manual versus non-manual occupation** | | | | | | | | | | | | | | |
| --- | --- | --- | --- | --- | --- | --- | --- | --- | --- | --- | --- | --- | --- | --- | --- |
|  | **Uncontrolled** | | |  | **Model 1** | | |  | **Model 2** | | |  | **Model 3** | | |
|  | ^a^ **Coef**  **(95%^b^ CI)** | | **P for trend** ^c^ |  | **Coef**  **(95% CI)** | | **P for trend** ^c^ |  | **Coef**  **(95% CI)** | | **P for trend** ^c^ |  | **Coef**  **(95% CI)** | | **P for trend** ^c^ |
| **Girls N=5,647** |  |  |  |  |  |  |  |  |  |  |  |  |  |  |  |
| BMI z-score at birth | 0.03 | (-0.02, 0.08) | 0.22 |  | 0.03 | (-0.02, 0.08) | 0.20 |  | 0.03 | (-0.02, 0.08) | 0.20 |  | 0.03 | (-0.02, 0.07) | 0.30 |
| BMI z-score trajectory from:  0 - 3 months | -0.04 | (-0.28, 0.19) | 0.72 |  | -0.08 | (-0.32, 0.16) | 0.52 |  | -0.10 | (-0.34, 0.14) | 0.42 |  | -0.06 | (-0.3, 0.18) | 0.64 |
| >3 - 12 months | -0.06 | (-0.14, 0.01) | 0.10 |  | -0.05 | (-0.13, 0.03) | 0.21 |  | -0.05 | (-0.12, 0.03) | 0.23 |  | -0.05 | (-0.13, 0.03) | 0.22 |
| >12 - 34 months | 0.00 | (-0.04, 0.04) | 0.90 |  | 0.00 | (-0.04, 0.04) | 0.95 |  | 0.00 | (-0.04, 0.04) | 0.99 |  | 0.00 | (-0.04, 0.04) | 0.90 |
| >34 - 84 months | 0.03 | (0.01, 0.05) | 0.001 |  | 0.03 | (0.01, 0.05) | 0.005 |  | 0.03 | (0.01, 0.05) | 0.01 |  | 0.02 | (0, 0.04) | 0.02 |
|  |  |  |  |  |  |  |  |  |  |  |  |  |  |  |  |
| **Boys N=6,043** |  |  |  |  |  |  |  |  |  |  |  |  |  |  |  |
| BMI z-score at birth | 0.03 | (-0.02, 0.07) | 0.28 |  | 0.03 | (-0.02, 0.07) | 0.29 |  | 0.03 | (-0.02, 0.07) | 0.24 |  | 0.03 | (-0.02, 0.08) | 0.20 |
| BMI z-score trajectory from:  0 - 3 months | 0.19 | (-0.05, 0.44) | 0.12 |  | 0.18 | (-0.06, 0.43) | 0.15 |  | 0.15 | (-0.1, 0.39) | 0.24 |  | 0.13 | (-0.12, 0.38) | 0.30 |
| >3 - 12 months | -0.02 | (-0.11, 0.06) | 0.56 |  | -0.01 | (-0.1, 0.07) | 0.76 |  | -0.01 | (-0.09, 0.07) | 0.86 |  | 0.00 | (-0.09, 0.08) | 0.95 |
| >12 - 34 months | -0.04 | (-0.09, 0) | 0.05 |  | -0.04 | (-0.09, 0) | 0.05 |  | -0.04 | (-0.09, 0) | 0.04 |  | -0.04 | (-0.08, 0) | 0.06 |
| >34 - 84 months | 0.04 | (0.02, 0.06) | <0.001 |  | 0.04 | (0.02, 0.06) | <0.001 |  | 0.04 | (0.02, 0.06) | <0.001 |  | 0.04 | (0.01, 0.06) | 0.001 |

Model 1: controlled for study trial arm, urban or rural location and East or West of Belarus

Model 2: as Model 1 additionally controlled for parents’ BMI

Model 3: as Model 2 additionally controlled for maternal smoking (never, ever or unknown) and older siblings (none, 1 or >1)

^a^ Coef=coefficient

^b^ CI=confidence interval

^c^ Trend across exposure categories

# **Web Table 7 Mean BMI z –score with imputation per increase in category of maternal education, N=16,861 (estimated from multilevel models)**

|  | **Mean difference in BMI-for-age z-score per category of maternal education** | | | | | | | | | | | | | | |
| --- | --- | --- | --- | --- | --- | --- | --- | --- | --- | --- | --- | --- | --- | --- | --- |
|  | **Uncontrolled** | | |  | **Model 1** | | |  | **Model 2** | | |  | **Model 3** | | |
|  | **Coef**^a^  **(95% CI**^b^**)** | | **P for trend** ^c^ |  | **Coef**  **(95% CI)** | | **P for trend** ^c^ |  | **Coef**  **(95% CI)** | | **P for trend** ^c^ |  | **Coef**  **(95% CI)** | | **P for trend** ^c^ |
| **Girls N=8,139** |  |  |  |  |  |  |  |  |  |  |  |  |  |  |  |
| BMI z-score at birth | 0.07 | (0.04, 0.09) | <0.001 |  | 0.05 | (0.02, 0.08) | <0.001 |  | 0.05 | (0.02, 0.07) | 0.003 |  | 0.03 | (0, 0.06) | 0.026 |
| BMI z-score trajectory from:  0 - 3 months | -0.26 | (-0.41, -0.11) | 0.001 |  | -0.20 | (-0.35, -0.05) | 0.008 |  | -0.20 | (-0.34, -0.05) | 0.01 |  | -0.16 | (-0.31, -0.01) | 0.04 |
| >3 - 12 months | -0.04 | (-0.08, 0.01) | 0.15 |  | -0.02 | (-0.06, 0.03) | 0.51 |  | -0.02 | (-0.06, 0.03) | 0.54 |  | -0.01 | (-0.06, 0.04) | 0.71 |
| >12 - 34 months | 0.00 | (-0.03, 0.03) | 0.96 |  | 0.00 | (-0.04, 0.03) | 0.78 |  | 0.00 | (-0.04, 0.03) | 0.81 |  | 0.00 | (-0.04, 0.03) | 0.81 |
| >34 - 84 months | 0.02 | (0.01, 0.04) | 0.004 |  | 0.02 | (0, 0.03) | 0.03 |  | 0.01 | (0, 0.03) | 0.09 |  | 0.01 | (0, 0.03) | 0.08 |
|  |  |  |  |  |  |  |  |  |  |  |  |  |  |  |  |
| **Boys N=8,722** |  |  |  |  |  |  |  |  |  |  |  |  |  |  |  |
| BMI z-score at birth | 0.06 | (0.04, 0.09) | <0.001 |  | 0.05 | (0.02, 0.08) | <0.001 |  | 0.05 | (0.02, 0.08) | 0.001 |  | 0.05 | (0.02, 0.07) | 0.002 |
| BMI z-score trajectory from:  0 - 3 months | -0.16 | (-0.32, -0.01) | 0.03 |  | -0.11 | (-0.26, 0.04) | 0.16 |  | -0.12 | (-0.27, 0.04) | 0.14 |  | -0.11 | (-0.26, 0.05) | 0.17 |
| >3 - 12 months | -0.01 | (-0.06, 0.04) | 0.66 |  | 0.01 | (-0.04, 0.06) | 0.72 |  | 0.01 | (-0.04, 0.06) | 0.64 |  | 0.02 | (-0.03, 0.08) | 0.37 |
| >12 - 34 months | -0.02 | (-0.05, 0.01) | 0.25 |  | -0.02 | (-0.05, 0.01) | 0.11 |  | -0.03 | (-0.06, 0) | 0.09 |  | -0.03 | (-0.06, 0) | 0.05 |
| >34 - 84 months | 0.03 | (0.01, 0.04) | 0.001 |  | 0.02 | (0.01, 0.04) | 0.002 |  | 0.02 | (0.01, 0.04) | 0.004 |  | 0.02 | (0.01, 0.04) | 0.004 |

Model 1: controlled for study trial arm, urban or rural location and East or West of Belarus

Model 2: as Model 1 additionally controlled for both parents’ BMI

Model 3: as Model 2 additionally controlled for maternal smoking (never, ever or unknown) and older siblings (none, 1 or >1)

^a^ Coef=coefficient

^b^ CI=confidence interval

^c^ Trend across exposure categories

# **Web Table 8 Mean BMI z –score with imputation per one unit increase in category of paternal education, N=16,861 (estimated from multilevel models)**

|  | **Difference in BMI z-score per category of paternal education** | | | | | | | | | | | | | | |
| --- | --- | --- | --- | --- | --- | --- | --- | --- | --- | --- | --- | --- | --- | --- | --- |
|  | **Uncontrolled** | | |  | **Model 1** | | |  | **Model 2** | | |  | **Model 3** | | |
|  | ^a^ **Coef**  **(95%^b^ CI)** | | **P for trend** ^c^ |  | **Coef**  **(95% CI)** | | **P for trend** ^c^ |  | **Coef**  **(95% CI)** | | **P for trend** ^c^ |  | **Coef**  **(95% CI)** | | **P for trend** ^c^ |
| **Girls N=8,139** |  |  |  |  |  |  |  |  |  |  |  |  |  |  |  |
| BMI z-score at birth | 0.05 | (0.02, 0.08) | 0.001 |  | 0.04 | (0.01, 0.07) | 0.008 |  | 0.03 | (0, 0.06) | 0.03 |  | 0.03 | (0, 0.06) | 0.05 |
| BMI z-score trajectory from:  0 - 3 months | -0.26 | (-0.41, -0.12) | <0.001 |  | -0.22 | (-0.37, -0.07) | 0.003 |  | -0.22 | (-0.37, -0.07) | 0.004 |  | -0.20 | (-0.35, -0.05) | 0.01 |
| >3 - 12 months | -0.01 | (-0.06, 0.04) | 0.68 |  | 0.01 | (-0.04, 0.06) | 0.63 |  | 0.01 | (-0.03, 0.06) | 0.54 |  | 0.02 | (-0.03, 0.06) | 0.53 |
| >12 - 34 months | 0.00 | (-0.03, 0.03) | 0.99 |  | 0.00 | (-0.04, 0.03) | 0.81 |  | 0.00 | (-0.04, 0.03) | 0.87 |  | 0.00 | (-0.03, 0.03) | 0.93 |
| >34 - 84 months | 0.02 | (0, 0.03) | 0.03 |  | 0.01 | (0, 0.02) | 0.18 |  | 0.01 | (-0.01, 0.02) | 0.29 |  | 0.01 | (-0.01, 0.02) | 0.38 |
|  |  |  |  |  |  |  |  |  |  |  |  |  |  |  |  |
| **Boys N=8,722** |  |  |  |  |  |  |  |  |  |  |  |  |  |  |  |
| BMI z-score at birth | 0.04 | (0.01, 0.07) | 0.01 |  | 0.03 | (0, 0.06) | 0.05 |  | 0.03 | (0, 0.06) | 0.06 |  | 0.03 | (0, 0.06) | 0.05 |
| BMI z-score trajectory from:  0 - 3 months | -0.10 | (-0.25, 0.05) | 0.21 |  | -0.07 | (-0.23, 0.08) | 0.36 |  | -0.08 | (-0.23, 0.08) | 0.33 |  | -0.08 | (-0.23, 0.07) | 0.31 |
| >3 - 12 months | -0.04 | (-0.09, 0.01) | 0.12 |  | -0.02 | (-0.07, 0.03) | 0.41 |  | -0.01 | (-0.07, 0.04) | 0.58 |  | -0.01 | (-0.06, 0.04) | 0.69 |
| >12 - 34 months | -0.03 | (-0.06, 0.01) | 0.13 |  | -0.03 | (-0.06, 0) | 0.07 |  | -0.03 | (-0.07, 0) | 0.07 |  | -0.03 | (-0.07, 0) | 0.06 |
| >34 - 84 months | 0.03 | (0.02, 0.04) | <0.001 |  | 0.03 | (0.01, 0.04) | <0.001 |  | 0.03 | (0.01, 0.04) | <0.001 |  | 0.03 | (0.01, 0.04) | <0.001 |

Model 1: controlled for length or height, study trial arm, urban or rural location and East or West of Belarus

Model 2: as Model 1 additionally controlled for parents’ BMI

Model 3: as Model 2 additionally controlled for maternal smoking (never, ever or unknown) and older siblings (none, 1 or >1)

^a^ Coef=coefficient

^b^ CI=confidence interval

^c^ Trend across exposure categories

# **Web Table 9 Mean BMI z –score with imputation per one unit increase in category of highest household occupation, N=16,861 (estimated from multilevel models)**

|  | **Difference in BMI z-score, manual versus non-manual occupation** | | | | | | | | | | | | | | |
| --- | --- | --- | --- | --- | --- | --- | --- | --- | --- | --- | --- | --- | --- | --- | --- |
|  | **Uncontrolled** | | |  | **Model 1** | | |  | **Model 2** | | |  | **Model 3** | | |
|  | ^a^ **Coef**  **(95%^b^ CI)** | | **P for trend** ^c^ |  | **Coef**  **(95% CI)** | | **P for trend** ^c^ |  | **Coef**  **(95% CI)** | | **P for trend** ^c^ |  | **Coef**  **(95% CI)** | | **P for trend** ^c^ |
| **Girls N=8,139** |  |  |  |  |  |  |  |  |  |  |  |  |  |  |  |
| BMI z-score at birth | 0.06 | (0.02, 0.1) | 0.003 |  | 0.06 | (0.02, 0.1) | 0.004 |  | 0.05 | (0.01, 0.09) | 0.019 |  | 0.05 | (0.01, 0.09) | 0.019 |
| BMI z-score trajectory from:  0 - 3 months | -0.19 | (-0.39, 0.01) | 0.07 |  | -0.21 | (-0.42, -0.01) | 0.04 |  | -0.21 | (-0.41, 0) | 0.05 |  | -0.19 | (-0.4, 0.01) | 0.07 |
| >3 - 12 months | -0.05 | (-0.12, 0.02) | 0.13 |  | -0.03 | (-0.1, 0.04) | 0.36 |  | -0.03 | (-0.09, 0.04) | 0.42 |  | -0.03 | (-0.1, 0.04) | 0.38 |
| >12 - 34 months | -0.02 | (-0.06, 0.02) | 0.38 |  | -0.02 | (-0.07, 0.02) | 0.33 |  | -0.02 | (-0.06, 0.02) | 0.36 |  | -0.02 | (-0.06, 0.03) | 0.43 |
| >34 - 84 months | 0.04 | (0.03, 0.06) | <0.001 |  | 0.04 | (0.02, 0.06) | <0.001 |  | 0.04 | (0.02, 0.06) | <0.001 |  | 0.04 | (0.02, 0.05) | <0.001 |
|  |  |  |  |  |  |  |  |  |  |  |  |  |  |  |  |
| **Boys N=8,722** |  |  |  |  |  |  |  |  |  |  |  |  |  |  |  |
| BMI z-score at birth | 0.04 | (0, 0.08) | 0.07 |  | 0.03 | (-0.01, 0.07) | 0.1 |  | 0.03 | (-0.01, 0.07) | 0.18 |  | 0.04 | (0, 0.08) | 0.07 |
| BMI z-score trajectory from:  0 - 3 months | 0.11 | (-0.1, 0.32) | 0.30 |  | 0.12 | (-0.09, 0.33) | 0.28 |  | 0.11 | (-0.1, 0.32) | 0.32 |  | 0.07 | (-0.14, 0.28) | 0.52 |
| >3 - 12 months | -0.04 | (-0.11, 0.03) | 0.31 |  | -0.02 | (-0.09, 0.05) | 0.57 |  | -0.01 | (-0.09, 0.06) | 0.7 |  | -0.01 | (-0.08, 0.07) | 0.85 |
| >12 - 34 months | -0.04 | (-0.08, 0) | 0.04 |  | -0.05 | (-0.09, -0.01) | 0.03 |  | -0.05 | (-0.09, -0.01) | 0.02 |  | -0.05 | (-0.09, -0.01) | 0.02 |
| >34 - 84 months | 0.05 | (0.03, 0.07) | <0.001 |  | 0.05 | (0.03, 0.06) | <0.001 |  | 0.04 | (0.03, 0.06) | <0.001 |  | 0.04 | (0.02, 0.06) | <0.001 |

Model 1: controlled for study trial arm, urban or rural location and East or West of Belarus

Model 2: as Model 1 additionally controlled for parents’ BMI

Model 3: as Model 2 additionally controlled for maternal smoking (never, ever or unknown) and older siblings (none, 1 or >1)

^a^ Coef=coefficient

^b^ CI=confidence interval

^c^ Trend across exposure categories

# **eMethods1. Estimating absolute differences in BMI z-scores**

*Models such as those presented in Table 2 were used to estimate absolute differences in BMI z-score between the highest and lowest categories of maternal education at age 7 years, using the lincom command in STATA and the following formula:*

*β_0_ = coefficient at birth*

*β_1_ = coefficient 0-3 months*

*β_2_ = coefficient 3-12 months*

*β_3_ = coefficient 12-34 months*

*β_4_ = coefficient 34-84 months*

*β_0_*2 + β_1_*0.25*2 + β_2_* 0.75*2 + β_3_*1.8*2 + β_4_*4.2*2.*

*Note:*

*1. The coefficients represent the interaction between maternal education and each age period.*

*2. Maternal education was analysed as one of three categories. In this model, the socioeconomic indicator is fitted as a continuous variable, so we assume a jump of two units from the lowest to the highest category of maternal education.*

*3. The numbers 0.25, 0.75, 1.8, and 4.2 represent the age period over which the difference in BMI z-score applies.*

# **eMethods2. Multiple imputation of missing values**

In a sensitivity analysis, we investigated whether loss to follow-up influenced the results by undertaking multiple imputation (MI) to generate values of missing outcomes and thereby including all 16,861 randomized participants in the multi-level model. We also generated values of any missing covariates of the analysis models. We imputed the missing values using chained equations imputation, which is a flexible imputation approach able to handle different variable types (i.e., continuous, binary, unordered categorical and ordered categorical). To ensure the imputed data maintained the same longitudinal structure of the observed data (i.e., capture the correlations between measurements at different time points), we generated distinct variables for all measurement time points and included all of these variables in the same imputation model. We note, there were no problems with collinearity. We also included, in the imputation model, all covariates of the analysis models, and predictors of missingness and the missing values. Using the *ice* command in STATA, we generated 20 imputed data sets on all PROBIT participants with at least 2 measurements (n = 16,861). Because the analysis models were fitted to males and females separately we imputed the data separately by sex. We analysed each imputed dataset separately, as per the non-imputed analysis, and combined the multiple sets of results using Rubin’s rules [Rubin DB. *Multiple Imputation for Nonresponse in Surveys*. Wiley: New York, 1987.].

| **Variable** | | | **Type of variable** | **N complete values** | **N imputed values** |
| --- | --- | --- | --- | --- | --- |
| Study trial arm | | | Dichotomous | 16861 | 0 |
| Urban or rural location | | | Dichotomous | 16861 | 0 |
| East or West of Belarus | | | Dichotomous | 16861 | 0 |
| Maternal smoking status | | | Nominal | 16861 | 0 |
| Maternal education | | | Nominal | 16861 | 0 |
| Paternal education | | | Nominal | 16280 | 581 |
| Highest household occupation | | | Dichotomous | 15621 | 1240 |
| Maternal BMI | | | Continuous | 13664 | 3197 |
| Paternal BMI | | | Continuous | 12457 | 4404 |
| Number of older children in the household | | | Ordinal | 16860 | 1 |
| BMI z-score at: | | birth | Continuous | 16847 | 14 |
|  | | 1m | Continuous | 16169 | 692 |
|  | | 2m | Continuous | 15969 | 892 |
|  | | 3m | Continuous | 16314 | 547 |
|  | | 6m | Continuous | 16192 | 669 |
|  | | 9m | Continuous | 15569 | 1292 |
|  | | 12m | Continuous | 14358 | 2503 |
|  | | 2.3-3.3y | Continuous | 7251 | 9610 |
|  | | at 6.5y | Continuous | 12771 | 4090 |
| Age at clinic visit at: | | 1m | Continuous | 16169 | 692 |
|  | | 2m | Continuous | 15969 | 892 |
|  | | 3m | Continuous | 16314 | 547 |
|  | | at 6m | Continuous | 16192 | 669 |
|  | | at 9m | Continuous | 15569 | 1292 |
|  | | 12m | Continuous | 14358 | 2503 |
|  | | 2.3-3.3y | Continuous | 7251 | 9610 |
|  | | 6.5y | Continuous | 12771 | 4090 |
| **Additional variables included only in MI model** | | | | | |
| Maternal age |  | | Continuous | 16861 | 0 |
| Paternal age |  | | Continuous | 16243 | 618 |
| Paternal smoking at 6.5y | | | Ordinal | 12569 | 4292 |
